# The Audiphone

**Published:** 1880-03

**Authors:** J. Allen Osmun

**Affiliations:** Newark, N. J.


ARTICLE IV.

### THE AUDIPHONE.

BY J. ALLEN OSMUN, M. D., NEWARK, N. J.

As the audiphone is destined to play so important a part in enabling those afflicted with deafness to hear distinctly, and since it is used in connection with the teeth, it seems eminently proper that the subject should receive the attention of the dental profession. This instrument is shaped like a square Japanese fan, and is made of a composition the major portion of which is vulcanite; at the back of this square portion is a cord, stretching from the upper edge to the handle. By means of this cord the instrument is tuned like a violin, and the tension is regulated according to the distance the sound has to travel. The upper edge of this audiphone is placed against the upper teeth, and the vibrations received on its surface are conveyed by the medium of the teeth to the acoustic nerves, and produce upon them an action similar to that produced by sound upon the drum of the ear. Now, the question arises in cases of deafness, where the persons have had the misfortune to lose their natural teeth, what denture will best supply their loss?

Having instituted a series of experiments, to test the merits of each, I submit them to the reader.

A lady, who had been deaf for nearly twenty years, called at my office to obtain a new denture, which would enable her to hear better with the audiphone. The one she was using was on silver, and was ill-fitting. Believing that accuracy of fit was the all-essential point, I naturally advised vulcanite. She, however, had been urged by several of the profession to wear gold, on account of being a good conductor of sound. Consequently, I made her a set on each base for trial, the gold plate fitting as well as was possible. She reports as follows: "I can hear very well with the silver plate; better with the gold; but with the vulcanite set the tones are much louder, and I can hear at a greater distance." Not being satisfied with a single trial, I repeated it in case of a partial denture, with same results.

With these experiments before us, together with the fact that with the natural teeth there is no medium to conduct sound, excepting the vibrations of the audiphone in connection with the teeth, the argument that a metal plate will facilitate hearing is untenable.

